# Death of a Hospital Treasurer

**Published:** 1915-07-31

**Authors:** 


### Death of a Hospital Treasurer.

By the sudden death at the age of eighty-one of Mr. Samuel Francis Stone, D.L., J.P., the Leicester Royal Infirmary has lost not only its hon. treasurer, but one of the oldest governors, and certainly the father of the present board. Mr. Stone attended a meeting of the house committee of the institution on Wednesday, July 14, and seemed in good health; but died of heart failure on the following Sunday night, just after retiring to bed. We have known Mr. Stone and his work for forty-five years. We mourn his loss, but are cheered by his example. Mr. Stone's first association with the infirmary was in 1859, when he became an annual governor. In the year 1876 he was appointed a member of the board of the institution, and in March 1902 was appointed treasurer in succession to Mr. William Unwin Heygate. Mr. Stone filled many offices in connection with the town and county of Leicester. A son of the late Mr. Samuel Stone, formerly town clerk, and the author of the well-known "Stone's Justices' Manual," he adopted the legal profession, and "took out his certificate" as a solicitor some sixty years ago. He was the senior partner of a well-known firm, from which he retired twenty years ago. Since then he has devoted much time and leisure to the advantage and welfare of the public. His sympathy with the relief of sickness and distress was shown by his long and close connection with the administration of the infirmary, to which he was a generous and timely contributor. On the occasion of his eightieth birthday on January 11, 1914, he handed to Sir Edward Wood a donation of £100 for the funds of the Children's Hospital. As one of the trustees of the charity of the late Mr. Benjamin Sutton, Mr. Stone attended with striking regularity the Saturday morning meetings of the trustees, over which his old friend, relative, and colleague, Mr. Thomas Fielding Johnson, a former chairman of the board of the infirmary, presided. He leaves three sons and two daughters. Two of the former are at present serving their country, one in the Army and the other in the Navy.

